# Communicating Guatemalan Villagers’ Experience of Aging Through Photography of Local Aging Advocates

**DOI:** 10.1093/geroni/igaa057.3072

**Published:** 2020-12-16

**Authors:** Margaret Perkinson

**Affiliations:** University of Hawaii, Honolulu, Hawaii, United States

## Abstract

What does it mean to grow old in a remote Guatemalan village? How do locals perceive the experience and meaning of later life, and how can a non-local access those perceptions? Volunteers from the village church’s committee on aging learned PhotoVoice methodology to document townspeople’s experiences of aging. Team members reassembled to discuss reasons for selecting a particular subject, how the photos’ contents related to their own lives, what the images said about aging in their village, and possible actions to consider in response. Qualitative analysis of the discussions revealed perceptions and meanings attached to family relationships, living environments, work and leisure activities, and health issues of their older subjects. The images evoked exchanges, commentary, and reflections among group members, creating an “eliciting context” in which processes of negotiated, shared meaning-making regarding later life emerged.

